# Cardiac Function and Iron Chelation in Thalassemia Major and Intermedia: a Review of the Underlying Pathophysiology and Approach to Chelation Management

**DOI:** 10.4084/MJHID.2009.002

**Published:** 2009-07-18

**Authors:** Athanasios Aessopos, Vasilios Berdoukas

**Affiliations:** First Dept. of Internal Medicine, University of Athens Medical School, Laiko Hospital, Athens, Greece

## Abstract

Heart disease is the leading cause of mortality and one of the main causes of morbidity in beta-thalassemia. Patients with homozygous thalassemia may have either a severe phenotype which is usually transfusion dependent or a milder form that is thalassemia intermedia. The two main factors that determine cardiac disease in homozygous β thalassemia are the high output state that results from chronic tissue hypoxia, hypoxia-induced compensatory reactions and iron overload. The high output state playing a major role in thalassaemia intermedia and the iron load being more significant in the major form. Arrhythmias, vascular involvement that leads to an increased pulmonary vascular resistance and an increased systemic vascular stiffness and valvular abnormalities also contribute to the cardiac dysfunction in varying degrees according to the severity of the phenotype. Endocrine abnormalities, infections, renal function and medications can also play a role in the overall cardiac function. For thalassaemia major, regular and adequate blood transfusions and iron chelation therapy are the mainstays of management. The approach to thalassaemia intermedia, today, is aimed at monitoring for complications and initiating, timely, regular transfusions and/or iron chelation therapy. Once the patients are on transfusions, then they should be managed in the same way as the thalassaemia major patients. If cardiac manifestations of dysfunction are present in either form of thalassaemia, high pre transfusion Hb levels need to be maintained in order to reduce cardiac output and appropriate intensive chelation therapy needs to be instituted. In general recommendations on chelation, today, are usually made according to the Cardiac Magnetic Resonance findings, if available. With the advances in the latter technology and the ability to tailor chelation therapy according to the MRI findings as well as the availability of three iron chelators, together with increasing the transfusions as need, it is hoped that the incidence of cardiac dysfunction in these syndromes will be markedly reduced. This of course depends very much on the attention to detail with the monitoring and the cooperation of the patient with both the recommended investigations and the prescribed chelation.

## Introduction:

β-thalassemia is an inherited hemoglobin disorder resulting from either homozygous or double heterozygous inheritance of two abnormal genes from the β – globin locus, leading to impaired synthesis of the β-globin chain and resulting in chronic dyserythropoietic anemia.^1^ Depending on the clinical severity, two forms, thalassemia major (TM) and thalassemia intermedia, (TI) are distinguished^1^. The majority of patients have TM but up to 25% may have TI.^1^ TM is rapidly fatal unless adequate transfusions, in conjunction with intensive iron chelation therapy, are started sufficiently early.^2^ In contrast, TI is generally characterized by a mild clinical picture, has a better prognosis and survival and requires therapeutic interventions only later in life, if at all.^3^ The clinical course of both forms of thalassemia (Th), if they remain untreated, is complicated by the multiple effects of chronic anaemia and of the resultant tissue hypoxia as well as by their compensatory reactions, including increased erythropoiesis with bone marrow expansion and increased intestinal iron absorption.^1^ Those manifestations are completely or partially inhibited nowadays in TM patients, due to the early application of regular transfusion-chelation therapy at the cost of chronic iron overload and the need for iron chelation therapy, while in TI patients these features are still present in varying degrees. Cardiovascular involvement represents a well-known complication and remains the primary cause of mortality both in TM and in TI.^2^–^4^ As discussed below, it seems quite different in the two forms of the disease. Despite the fact that both forms share common basic underlying pathophysiological mechanisms that affect the heart, the different degree of contribution of these mechanisms in TM and in TI, result in a variety of left and right heart involvement, which ultimately lead to congestive heart failure. Knowledge of the complexity of the underlying mechanisms in thalassaemia may help to prevent or to treat the heart injury.

## Common mechanisms of heart injury in Thalassemia (Th):

Cardiac structure and function in Th are mainly affected by two factors: iron load and increased cardiac output. Additional factors are involved and will be discussed.

### The cardiac iron load:

Iron overload results from two main mechanisms. In both TM and TI, it is associated with red cell transfusion and increased intestinal iron absorption^1^. The iron overload in TM is dominated by the transfusion iron, while in TI the absorption is the greatest source. Furthermore, the disease itself, including ineffective erythropoiesis as well as peripheral haemolysis results in selective tissue iron deposition. Therefore, although iron overload is mainly a problem of TM patients, it also exists to a lesser extent in TI. The heart, along with liver and endocrine glands, is one of the main organs where iron deposition causes severe complications.^5^ Iron overload interferes in the cardiomyocytes’ capacity to catalyze the formation of deleterious oxygen free radicals.^5^ The quantification of myocardial iron content is not generally easy and only T2* CMR has allowed a reliable estimation in a large number of TM patients.^6^ There are two mechanisms of iron related injury; these are direct and indirect.

### Direct Iron related injury:

In TM survival was dependent on regular transfusion. Patients receive between 0.3–0.5 mg/Kg/day of iron through transfusions. The average daily losses are less than 1mg in males and 2 mgs in menstruating females. There are no other physiological mechanisms for effecting body iron reduction; therefore the body stores the iron. Before the availability of iron chelation therapy, the majority of transfused TM patients died, usually in the second and third decade of life, from cardiac failure that was due to iron overload. In TI the increased gastro-intestinal absorption of iron, which is much higher than that in normal individuals is most likely due to a paradoxical suppression of hepcidin.^7^–^9^ In dyserythropoietic anaemias, this suppression has recently been found to be induced by Matriptase-2, a transmembrane serine protease.^10^ Hepcidin interferes in iron homeostasis by inhibiting iron absorption from duodenal enterocytes, iron release from hepatocytes and from macrophages that recycle iron from senescent erythrocytes.^7^,^8^ In Th, the accumulated iron, is thought to saturate liver firstly, and then to accumulate in other organs. Therefore In the less loaded TI, the absorbed iron seems to accumulate mainly in the liver and less frequently involves the heart. A number of studies using CMR T2* have demonstrated this finding. One study in 31 TI patients revealed that 23% of cases had cardiac iron overload, defined as a T2* value <20 msec^11^–^13^. The usual management of TI is clinical observation with occasional transfusions and intervention with regular transfusions and iron chelation therapy if indicated.In histological examination of the heart in patients with TM, the iron accumulates in all four chambers, papillary muscles and the electrical conduction system, including the sinoatrial and atrioventricular nodes. In the free wall of the left ventricle there is more iron concentrated in the epicardial layers than in the endocardial and middle third^14^. Iron is stored in cells, including myocytes, in the form of ferritin, haemosiderin and free iron. The latter is referred as the labile cellular iron (LCI).^15^ There is a significant flux between the three forms, with haemosiderin being the least soluble and accessible. The LCI is the most toxic form as it stimulates the formation of free radicals (Fenton Reactions), which results in peroxidative damage of membrane lipids and proteins provoking cellular injury. In heart, this leads to impaired function of the mitochondrial respiratory chain and is clinically manifested by reduction of cardiac muscular contractility and CCF development.^16^ To date, at least 90 genes that control iron metabolism have been identified.^17^ In each individual therefore, it is highly likely that the handling of iron and the action of iron chelators will be different. These concepts fit in well with the wide range of reported different clinical cardiac courses seen mainly in TM patients who have followed similar life-time, well accepted treatment.^18^ Knowledge derived by recent MRI studies which also assessed cardiac function, showed that all patients with reduced LV function had cardiac iron overload and in many cases this was severe.^6^,^19^,^20^ This strongly suggests that in addition to the damage caused by the accumulated iron, excessive iron in the myocytes results in greater amounts of LCI leading to free radical formation that overwhelms the antioxidant mechanisms and ultimately precipitates cardiac dysfunction. On the other hand, in the above MRI studies, despite heavy iron load, many TM patients maintained normal cardiac function, albeit perhaps temporarily, and, as discussed above, this may be related to their intracellular iron metabolism, in particular their handling of oxidants. It has been shown that TM individuals who had the genetic factor apo-lipoprotein E4 are at greater risk for LV dysfunction than those with other alleles such as apo E2 and apo E 3 because of reduced ability to handle oxidative stress.^21^,^22^

### Indirect iron related injury:

All the following factors related to indirect iron related cardiac injury are more common in TM than TI. However, they are relevant to both.

#### Infections:

Any significant infection may precipitate cardiac failure particularly in the presence of other underlying cardiac pathology. Immune competence in beta-thalassemia is impaired^23^–^26^ and patients are more vulnerable to infections. Furthermore, siderophore bacteria, such as yersinia and klebsiella, rely on iron for multiplication and grow well in the microenvironment of transfusion iron loaded patients^25^. Iron overload is considered to be the main etiologic factor that can disturb the immune balance in favour of the growth of infectious organisms.^24^ This may also be affected by differences in the existing immunogenetic profile in Th^26^ especially with respect to viral infections. Two severe cardiac complications, pericarditis and myocarditis, are linked to iron load induced viral infection susceptibility.Pericarditis was frequently seen in Th, In TM patients with poor or no chelation in the past^27^ it was quite frequent (50%). Today, with the use of chelation therapy, it is very rare (5%).^18^ Similarly, the reported myocarditis in TM with decreased LV function,^28^ seems most likely to be related to iron load. Even though there may be histological evidence of infections, as demonstrated by lymphocytic infiltration, recent CMR evidence shows that LV failure only occurs in the presence of excessive iron.^6^,^19^,^29^ Viral myocarditis without iron in the heart may be rare and may follow similar outcomes to those of the normal population.

#### Arrhythmias:

The iron induced cardiac toxicity is often complicated by arrhythmias such as extra atrial and ventricular beats, paroxysmal atrial tachycardia, flutter or fibrillation. The high output state may also be related to the incidence of arrhythmias to a lesser extent. Life threatening ventricular tachycardia is rare and often associated with reduced LV function. Short runs of non specific ventricular tachycardia are quite common and are more common with elevated cardiac iron^30^. Atrial arrhythmias occur more frequently in both TI and TM. These are more clinically relevant and difficult to treat. They do not necessarily relate to the degree of cardiac iron load at the time of onset, but may result from past damage caused by the iron load or high cardiac output. Some of these arrhythmias can also be triggering factors for CCF or reduced cardiac function in TM patients without previous obvious LV dysfunction.

#### Endocrine abnormalities:

Endocrine abnormalities occur in Th but with greater frequency in TM. Iron toxicity may also indirectly affect heart function by damaging other organs in varying degrees. The endocrine abnormalities hypothyroidism and diabetes mellitus can have a significant impact on cardiac function.^31^ Hypothyroidism can precipitate pericardial effusion, decreased LV function, bradycardia and increased peripheral vascular resistance. The onset of diabetes is often associated with the presentation of cardiac dysfunction. This correlates with a recent finding that pancreatic iron correlates well with cardiac iron and not with hepatic iron.^32^ Chronic hyperglycaemia is an oxidative stress on many organs, particularly the heart. Hypocalcaemia associated with occult or overt hypoparathyroidism can precipitate heart dysfunction.

#### Medications:

Vitamin C has been given to patients with in order to enhance their iron excretion when they are on chelation therapy. There have been case reports of TM who developed sudden acute cardiac failure with a fatal outcome that had been precipitated by the administration of Vitamin C possibly by releasing free iron that is toxic.^33^

#### Vascular Involvement (After load):

Systemic arterial involvement in Th, has been observed recently through clinical, functional^34^ and anatomical^35^ studies, and plays a role in the development of cardiac dysfunction by affecting heart after load. Vascular involvement starts early in life and becomes obvious in the older patients,^36^ principally in TI. Haemolysis participates in this injury as does iron overload, most likely through the effect of the labile plasma iron (LPI). The other contributory mechanisms will be discussed in detail below in the section on elastic tissue abnormalities.

### Increased Cardiac Output (CO) effect:

Disease related increased CO, resulting in increased workload on the heart, contributes to the development of cardiac dysfunction in Th patients. Anemia together with marrow expansion leads to volume overload that then demands increased contractility. (Starling’s Law).In normal individuals. Hb levels between 80–100 g/l do not have any effect on the resting cardiac output^37^,^38^. TM patients, however, even those well transfused (pre transfusion Hb level > 95 g/l) with excellent suppression of marrow activity and with a mean Hb level of 113 g/l, demonstrate some degree of high cardiac output (Cardiac Index 4.3±0.9/3.in TM cf. 3.8±0.8 P<.01 in normal individuals).^18^ In patients with high output state, the heart‘s systolic function index and ejection fraction is expected to be higher than in normal subjects. Thus, for Th patients, even well transfused TM, it has been recommended that a normal LVEF should be above 60%^39^,^40^ and the degree of CO increase should be taken into account when assessing EF in each individual patient^41^. In those TM who are poorly transfused the increased cardiac output will be greater. In TI, with minimal to no transfusion, the increased cardiac output represents one of the basic pathophysiologic mechanisms of cardiovascular involvement and is a constant finding.^42^,^43^,^44^,^45^ More specifically, echocardiographic measurements reveal an almost two-fold increase in cardiac output levels, compared to normal subjects (Table 1).^42^ Indications of the presence of high output state were also derived by a cardiac magnetic resonance imaging (CMR) study in TI patients.^9^ Chronic hemolytic anemia, resulting from ineffective erythropoiesis, is the hallmark of all thalassemia syndromes.^1^ In TI, chronic anemia, however, is not always severe (hemoglobin levels range usually between 70 and 110 g/l) and apparently is not the only cause of high output state in these patients. Besides the overall hemoglobin level, the proportions of the different hemoglobin types, especially the high percentage of fetal hemoglobin (HbF), are also important. More specifically, HbF reduces tissue oxygen delivery due to its increased oxygen affinity.^46^ Thus, both chronic anemia and increased HbF percentage result in prolonged tissue hypoxia. This in turn, leads to a compensatory bone marrow expansion, with extramedullary haemopoiesis, splenomegaly and hepatomegaly, all of which also contribute to the high output state through peripheral vasodilatation and shunt development.^42^, ^46^–^48^ Similarly and more impressively, compensatory mechanisms also occur in TM who are not adequately transfused. In addition, vessels in Th are more susceptible to pulse pressure-driven dilatation, due to a co-existent elastic tissue injury, which is discussed in details below. Liver iron load or viral induced hepatic injury can also contribute, as cirrhosis can increase CO significantly.^49^ The contribution of peripheral vasodilatation and intramedullary shunting seems to play an important role in the high output state. Indeed, it has been shown that the abolition of splenic shunting and the increase in hemoglobin level following splenectomy are not sufficient to counteract the preexistent high cardiac output levels in Th.^47^, ^48^

### Additonal Factors that impact on Cardiac Injury

#### Haemolysis-induced tissue injury – Vascular involvement and elastic tissue abnormalities:

Chronic haemolysis and iron overload, are currently considered as sources of strong oxidative stress. Reports have shown that the free haeme and the red cell membrane elements that are produced during haemolysis have a negative effect on nitric oxide and arginine availability, which in turn promotes vasoconstriction.^50^ At the same time, they lead to further endothelial dysfunction, resulting in a more pronounced nitric oxide reduction, as well as to a diffuse elastic tissue injury. The presence of such an elastic tissue defect has been described with a high prevalence in patients with haemoglobinopathies, especially in those with Th.^36^,^51^ The defect resembles hereditary pseudoxanthoma elasticum (PXE), a rare (1:70000 to 1:160000) connective tissue disorder, and covers the whole clinical spectrum of PXE, which consists mainly of skin (small yellowish papules or larger coalescent plaques), ocular and vascular manifestations (degeneration of the elastic lamina of the arterial wall, often with calcification).^36^,^51^ Endocardium, cardiac valves and pericardium may also be involved.^36^,^42^ As the clinical expression of the elastic tissue injury is age-related, TI patients are more affected by PXE lesions due to their prolonged survival. Thus, it has been shown that TI patients aged >30 years (mean age 41.4 years) presented a 55% occurrence of tibial artery calcification as part of elastic tissue abnormalities^52^. Interestingly, histopathological studies in TI have shown it to be present in removed spleens even from the first decade of life.^35^ On the other hand, the degenerative arterial lesions observed in the elastic lamina and adventitia render vessels more susceptible to dilatation by pulse pressure increase. Finally, the functional component of the arterial involvement was recently studied in TM, sickle-cell anemia and sickle-thalassemia patients. Increased arterial stiffness along with endothelial dysfunction was encountered and attributed to the two common pathogenic mechanisms, namely haemolysis and iron load.^34^,^53^

#### Valvular involvement:

Although valvular involvement is present in Th, it is more pronounced in TI. Endocardial degenerative lesions, in the form of thickening and calcification, affect the cardiac valves, mitral annulus and papillary muscles and this is often followed by moderate valvular regurgitation and occasionally by aortic stenosis. These findings were described echocardiographically with a high frequency in a large group of 110 TI patients.^42^ More specifically, leaflet thickening was present in 48% of patients, endocardial calcification in 21%, mitral regurgitation in 47%, aortic regurgitation in 15%, while there were 3 cases with mild to moderate aortic stenosis. The hyperkinetic state due to the high output, the iron overload and primarily the aforementioned elastic tissue abnormalities have been suggested as the responsible pathogenic mechanisms.^42^,^43^ Although the haemodynamic consequences of the above mild or moderate valvular abnormalities are not usually significant, they may have an additive effect when associated with the other pathogenic mechanisms in the development of heart disease. Moreover, atrioventricular conduction disturbances as well as the risk of cerebrovascular thrombotic events, in the context of a coexistent hypercoagulable state, may also play a role.^42^,^54^

#### Hypercoagulability:

Is a well-described entity in Th.^55^ A number of pathogenic mechanisms have been discussed in relation to the underlying genetic defect and its consequences, namely haemolysis and iron overload and the resulting oxidative tissue damage. More specifically, the free α-globin chains that result from the decreased synthesis of the β-chains, along with the free iron provoke oxidative damage to the red blood cell membrane proteins; these changes result in the exposure of negatively charged phospholipids, which create a pre-coagulant surface.^55^,^56^ Moreover, data derived from TM and sickling syndromes, as described above, showed that endothelial function is also impaired.^34^,^53^ Oxidative damage, resulting once again from the two common mechanisms, haemolysis and iron load, leads to an increase expression of adhesion molecules ICAM and VCAM and impaired NO bioavailability, hence provoking hypercoagulability and decreasing NO-dependent, flow-mediated dilatation.^34^,^57^ Furthermore, platelets are activated with enhanced aggregation, while splenectomy increases platelet counts and induces membranes abnormalities that enhanced the already increased platelet aggregation. In parallel, the observed deficiency of the coagulation inhibitors, protein C and protein S, the elevated levels of thrombin-ATIII complex due to splenectomy and/or liver dysfunction as well as the co-inheritance of several coagulation defects, such as factor V (Leiden) and factor II deficiency, may also contribute to the pathogenesis of hypercoagulability in thalassemia.^55^,^58^ Finally, a strong inflammatory reaction has been noticed, expressed by the elevated circulating levels of cytokines and adhesion molecules, and the monocyte and neutrophil activation, hence promoting hypercoagulability.^57^

## Cardiovascular Consequences

### Vascular manifestations:

The combination of hypercoagulability and haemolysis-related elastic tissue abnormalities may lead to a wide spectrum of vascular complications. The elastic tissue abnormalities, on one hand, have been associated with a number of vascular complications, which have been sporadically observed in Th patients. These findings include fatal cerebral haemorrhages, anginal symptoms, ascending aorta aneurysm formation and gastrointestinal bleeding.^54^,^59^,^60^ Elastic tissue abnormalities may also contribute to the frequently encountered leg ulcerations in TI patients and may explain the observed development of transfusion-induced arterial hypertension in sickle cell anaemia and β-thalassemia patients^61^,^62^. On the other hand, the thalassemia-related hypercoagulability, sometimes in combination with the elastic tissue defects, has been held responsible for a high frequency of thromboembolic complications. Thromboembolic events were encountered in two large cohorts of thalassemia patients, including both TM and TI, with a frequency of 4.3% και 5.2%, respectively.^63^,^64^ It is noteworthy that the prevalence of such events was higher in splenectomised patients than in non-splenectomised ones. In particular, thromboembolic complications were even more frequent in transfusion-independent splenectomised TI (29%), compared to regularly transfused TM (2%), a finding that emphasizes the role of transfusion therapy in the inhibition of hypercoagulability in thalassemia patients.^65^ Such events comprised deep vein thrombosis (40%), portal vein thrombosis (19%), pulmonary thromboembolism (12%), cerebral thrombosis (9%) as well as recurrent arterial occlusion and others (20%). A recently published multinational cohort comprising 8,860 thalassaemia patients from the Mediterranean region and Iran showed that thromboembolic events were 4.38 times more frequent in TI than in TM, and were particularly prevalent in splenectomised patients and patients with profound anaemia (haemoglobin level <90 g/l).^66^ Ischemic strokes have also been described in combination with cardiac valvular lesions – a consequence of the elastic tissue defect and/or atrial fibrillation^54^ on a background of hypercoagulability. At the same time, thrombosis may be a silent, subclinical process, as autopsy findings of thrombi in the microvasculature of lungs and brain have been described in the absence of clinical manifestations or other known risk factors.^67^

### Right heart involvement:

Right sided heart involvement in Th may result from both pulmonary hypertension (PHT) and severe iron overload. In well transfused and chelated TM, PHT is rare. It is however, presenting with increasing frequency in TM, particularly those who are poorly transfused and chelated, even at younger ages.^68^ Those, who are adequately transfused but who are poorly chelated, may present with dominant right sided heart involvement^69^ with hepatic distension and pain with minimal dyspnoea, without evidence of PHT. PHT represents a prominent complication in TI. Almost 60% of cases in a large cohort of 110 adult TI patients had developed PHT.^42^ More specifically, peak systolic tricuspid gradient values >30mmHg, indicative of pulmonary hypertension, were present in 59.1% of TI that was age related, while values >50 mmHg were present in 7.3% of cases. Additional reports confirmed the above finding,^70^–^72^ while a recent study that compared TI with TM showed that PHT is a typical feature of non-transfused TI and not a simple age-related effect due to their prolonged survival.^43^ PHT seems to be the leading cause of congestive heart failure in TI, due to the subsequent right heart insufficiency usually with maintenance of LV function. The combination of high output state and increased pulmonary vascular resistance has been held responsible for the development of PHT.^42^,^43^ It is more pronounced in TI than in well transfused TM. The increased pulmonary vascular resistance in β-thalassemia is multifactorial. The fact that most subtypes of chronic haemolytic anemia may develop pulmonary hypertension suggests that there is a pathogenic link between the two conditions.^71^ The role of chronic haemolysis in the development of PHT through the induction of nitric oxide and arginine deficiency, which promotes vasoconstriction, has been recently stressed.^50^ At the same time, as stated above, haemolysis has also been associated with the coexistent diffuse elastic tissue defect. Degenerative elastic tissue lesions have been encountered in pulmonary autopsies in patients with haemoglobinopathies, such as sickle cell disease.^73^ Moreover, endothelial dysfunction promotes hypercoagulability and *in situ* thrombus formation within the pulmonary vascular bed. In β-thalassemia, in particular, the oxidative stress resulting from chronic haemolysis is enhanced by the presence of iron overload and free-radical formation and the expected effect seem to be more pronounced. In addition, iron overload is associated with interstitial pulmonary fibrosis and may affect pulmonary vascular resistance.^72^ Hypercoagulability, as discussed above, is a well-described co-morbid state in β-thalassemia, especially in non-transfused TI patients. Extensive thromboembolic lesions have been found in the pulmonary arterioles of splenectomised thalassaemics in post-mortem autopsies, leading to the reduction of the total pulmonary vascular bed^67^. Lung infections, chest deformities intrathoracic extramedullary haemopoietic masses and transient LV dysfunction may also contribute to pulmonary vascular resistance.^42^ All the mechanisms for PHT development can be inhibited by adequate transfusion and chelation therapy and explain why that finding is a rare phenomenon in TM.

### Left ventricular involvement:

The main mechanism of left ventricular involvement in Th is iron overload and secondarily the increased cardiac output. The reduction in LVEF is a major element in TM for cardiomyopathy and the worst prognostic feature with respect to patient survival. In well transfused TM (pre transfusion Hb > 95g/l) the iron overload predominates and in TI the increased cardiac output is prominent. Furthermore, the resulting elevation of systemic impedance that is presented to left ventricle leads to a less favourable interaction between left ventricular ejection and systemic arterial compliance, which contributes to left ventricular impairment.^74^,^75^ These changes are aggravated by the advancing age. Besides peripheral vascular disorders, the coexistence of coronary artery involvement, infections related to iron load, endocrine abnormalities, arrhythmias and valvular lesions, render left ventricular function more susceptible to decompensation. Th are more likely to present with overt cardiac dysfunction in situations of stress, such as excessive physical activity or other conditions requiring increase cardiac work load as fever or significant anaemia. In situations of increased stress, particularly in TI, LV cardiac decompensation may present with sudden worsening of preexisting PHT due to further increase in pulmonary vascular resistance.

## Chelation treatment for prevention and treatment of iron induced heart disease

### Chelation therapy:

Comprehensive treatment of both TM and TI is beyond the scope of this review. The approach to prevention and reversal of cardiac disease is principally based on TM. However for both TM and TI (once the decision to transfuse is made), it is important to minimize the cardiac output with adequate levels of pre-transfusion Hb (> 95g/l in general and higher if there is evidence of PHT or marginal cardiac function) and to remove the iron. The monitoring to determine the degree of cardiac iron overload is by Cardiac Magnetic Resonance (CMR) T2* assessment. Chelation treatment today should be guided by MRI findings, if the technique is available. We are in a transient phase of knowledge with the availability of MRI and new chelating agents. Important questions with respect to best management to avoid iron induced cardiac disease remain to be elucidated. Optimal management may be clarified from results of different trials and current ongoing follow up studies from many subgroups of patients using different regimes.In the presence of excess cardiac and or hepatic iron, treatment strategies include increase of the dose and/or frequency of desferrioxamine, switch to oral chelators (deferiprone or deferasirox) or to the combination of deferiprone with desferrioxamine, provided there are no contraindications to their use.^76^,^77^ Combination of the two iron chelators (desferrioxamine and deferiprone) seems to maximize the efficacy producing additive and synergistic effects in iron excretion.^78^,^79^ It seems that each of those two agents chelates iron from different pools and there is at least an additive effect when combined treatment is administered.^80^

Available evidence now suggests that combined therapy should be the treatment of choice for patients with established cardiac failure. Continuous desferrioxamine infusions alone, have been shown to improve cardiac function and salvage patients^81^ and is the treatment of choice if combination therapy is contraindicated. We have reported two cases with severe CCF who reversed with intensive combination therapy^82^,^83^ and we have at least 8 more patients with similar outcome. Two other studies show similar responses.^84^,^85^ In a recent study with combined treatment, apart from significant reduction in ferritin, cardiac and liver iron and improvement in cardiac function, the absolute endothelial function was also improved^77^. Furthermore, improvement with glucose tolerance with the use of combination therapy has been reported^86^,^87^ as well as anecdotal reports of improvement in other endocrine functions.

With respect to hepatic iron removal, the efficacy of the two oral chelators is at least equal to the standard doses of desferrioxamine^20^,^88^,^89^. Recent and ongoing studies have demonstrated that deferiprone, a small molecule that permeates all tissues, is more efficient in removing cardiac iron and improving cardiac function than desferrioxamine.^20^,^90^,^91^ Some preliminary clinical and laboratory observations with deferasirox are encouraging with respect to removal of cardiac iron.^92^,^93^ As yet, there are no studies with combinations of deferasirox and desferrioxamine so this therapeutic regime cannot be recommended at this stage. According to the current knowledge and based on the CMR findings, the suggested chelation regimes are as follows:

### Acceptable Cardiac Iron:

For patients with T2* > 20 ms., the therapeutic strategy should be continuation of monotherapy with either desferrioxamine or either of the available oral chelators (deferiprone and desferasirox) with regular follow-up. For the patient’s convenience, desferrioxamine administration may be converted to either of the two oral chelators. If there has been iron overload in the past that was attributed to desferrioxamine therapy and that was subsequently cleared with intensification of chelation therapy, then monotherapy with desferrioxamine is not recommended.

### Mild to Moderate Cardiac Iron Loading:

T2* values between 10–20ms are considered to reflect a mild to moderately iron loaded myocardium. Bearing in mind that the patients may be at risk of developing cardiac problems under stress such as infections, clearing myocardial tissue from iron seems to be a rational target. Therefore, combined treatment for these patients should not be a priori excluded. Patients have presented with LV dysfunction at levels of T2* of 15 msec, without any precipitating factors.^19^,^77^ Therefore, if T2* is ≤ 15 msec, combination chelation therapy is recommended.^76^ However, questions still exist, regarding the frequency and the amount of desferrioxamine administration that is appropriate in a combined regimen. A dose of 35–40mg/kg/day three-four times weekly combined with deferiprone at a dose of 75mg/kg/day seems to be reasonable. In patients with T2* 15–20 ms, monotherapy with deferiprone and deferasirox are available options.^20^,^88^ However, in this circumstance close monitoring is necessary. Patients treated up to the time of the MRI with desferrioxamine in this category and who availed themselves of that treatment satisfactorily, in general should not be on monotherapy with that compound, as desferrioxamine was inadequate at preventing the iron accumulation in the heart and may indicate some type of resistance to its efficacy within that patient. If however, the patient’s adherence to treatment with it was poor, then it may be appropriate in higher doses and frequency, provided the patient can be convinced to use it.

### Heavy Cardiac Iron Load:

Patients with T2* <10msec are considered to have severe iron overload and this category includes most patients with reduced left ventricular (LV) function. Even those patients with normal ejection fraction in this category are considered to have a significant risk of developing cardiac dysfunction. Thus all patients in this category have a strong indication for combined chelation treatment. The doses of the two medications should be similar to those described for patients with CCF (see below) but with the desferrioxamine being given as a subcutaneous infusion. If deferiprone is contraindicated, then intensive intravenous continuous desferrioxamine infusions are the treatment of choice.

#### Heart Failure:

For patients with heart failure desferrioxamine should be administrated at a dose of 60–80mg/kg/day intravenously and deferiprone at a dose of 75–100mg/kg/day in three divided doses. If deferiprone is contraindicated, the patient should be managed with continuous desferrioxamine infusions, which usually require the placement of an indwelling catheter^94^. It seems however, that the rate of removal of iron with such therapy is much slower than with combination therapy.^77^ Caution should be taken with the 24h desferrioxamine infusion to avoid fluid overload especially when intravenous antibiotics and anti arrhythmic agents are also indicated.

### Treatment Modifications:

Any treatment modification should be followed by close monitoring. Should any serious adverse effect present as a consequence of the administration of a particular chelator, appropriate guidelines as to its continued use should be followed. If treatment has ultimately modified the MRI patient’s classification then, it may be adjusted as discussed above according to the changes in MRI values. In all of the above, hepatic iron and endocrine status should also be considered and modification to the recommended regimes should be made in order to achieve normal hepatic iron levels in the long term.

#### Guidelines if MRI is not available:

In situations in which MRI is not available, then all the patients’ traditional parameters need to be analysed, (ferritins, liver iron concentrations) as well as ECG and echocardiogram. These may serve as a guide to treatment. Furthermore, according to knowledge from MRI studies, all patients with reductions in LVEF have excessive cardiac iron load. Any echocardiographic evidence of reduce cardiac function should be considered as being associated with excessive cardiac iron load and should be managed accordingly. In countries where follow up of patients has become available and who had been treated with desferrioxamine, up to 65% of patients have cardiac iron load. In Sardinia, 13% had severe cardiac iron overload.^29^ In our study 48% of patients have T2* < 15 ms^19^. In countries were patients’ compliance to treatment is inadequate, there was poor availability of chelation and/or the follow up was not well organized, the percentage of cardiac iron loaded patients is likely to be higher. Therefore, for patients who have never had optimal care, it is very likely the patients will have cardiac iron load and intensive combination chelation is the treatment of choice. In patients who have been poorly chelated, the risk of chelation toxicity is minimal and would only be likely to occur after prolonged therapy, however, it is important to be vigilant for such complications. If compliance with desferrioxamine has been an issue, as evidenced by high ferritin or hepatic iron, then either of the two available oral chelators is appropriate therapy. MRI is more necessary for those patients, who have had good chelation therapy with desferrioxamine but who are at risk of chelation inadequacy with respect to the heart and for those who have had treatment modification in order to follow the efficacy of the changed chelation regime.

## Conclusions on Heart Disease:

This formerly catastrophic genetic defect has been revolutionized with the availability of adequate chelation therapy and more recently with other important advances particularly MRI. Iron related heart failure is reversible in TM provided appropriate interventions are made in a timely manner. It should no longer be considered a terminal event and intensive attention to the parameters mentioned above can result in complete reversal with markedly improved quality of life.It remains important, practically, to aim to maintain low LIC’s and ferritin levels in Th (both TM and TI), particularly as the latter are easily accessible and assessable. Similarly, echocardiography should remain a routine tool as it does have some predictive value and can also be used to monitor patients in whom intensification of chelation therapy has been instituted.CMR can be particularly helpful in identifying all TM patients at risk of developing heart disease by assessing the cardiac iron load. Chelation therapy can be tailored to remove the excess heart iron. Attention to patient’s continuous compliance with adequate chelation is mandatory.The definite ability to know and reduce cardiac iron as well as improvement in cardiac function that can be achieved by appropriate chelation, should certainly lead to even further significant reduction in cardiac mortality and morbidity.

## Figures and Tables

**Table 1.** Data derived from a cohort previously published by Aessopos et al.^2^

	**Patients** (n=110)	**Controls** (n=76)	***p***
Right ventricular diameter (mm)	23±4	19±3	<.001
Left atrial diameter (mm)	42±6	33±3	<.001
Left ventricular end-diastolic diameter (mm)	55±5	48±2	<.001
Left ventricular end-systolic diameter (mm)	32±4	27±2	<.001
Interventricular septum thickness (mm)	9.7±1.0	8.6±0.9	<.001
Posterior wall thickness (mm)	9.6±1.0	8.5±0.8	<.001
Left ventricular mass index (g/m^2^)	126±30	86±10	<.001
Shortening fraction (%)	43±5	44±3	NS
Ejection fraction (%)	73±6	75±3	<.05
Cardiac index (l/min/m2)	5.45±1.33	3.82±0.80	<.001
Peak early transmitral diastolic velocity - E (cm/sec)	99±20	80±15	<.001
Peak late transmitral diastolic velocity – A (cm/sec)	69±19	58±12	<.001
E/A	1.51±0.48	1.42±0.34	NS
E deceleration time (msec)	152±34	164±33	NS
Isovolumic relaxation time (msec)	47±15	51±10	NS
Peak systolic tricuspid gradient (mmHg)	33.15±14.06	20.77±4.23	<.001
Total pulmonary resistance (dynes · sec · cm^−5^)	451±294	245±93	<.001
